# Value of Percutaneous Transhepatic Gallbladder Drainage for Advanced Acute Cholecystitis as a Bridging Procedure: A Single-Center Retrospective Study

**DOI:** 10.3390/jcm14227955

**Published:** 2025-11-10

**Authors:** Benoit Geng, Raffaella Sguinzi, Alexis Litchinko, Benoît Gremaud, Philippe Froment, Michel Adamina

**Affiliations:** 1 Department of Surgery, Cantonal Hospital of Fribourg, Chemin des Pensionnats 2/6, 1752 Villars-sur-Glâne, Switzerland; 2 Department of Medical and Surgical Specialties, Faculty of Science and Medicine, University of Fribourg, 1700 Fribourg, Switzerland; 3 Department of Visceral Surgery and Medicine, Inselspital, Bern University Hospital, 3010 Bern, Switzerland

**Keywords:** acute cholecystitis, gallbladder drainage, percutaneous drainage, cholecystectomy, laparoscopic, biliary decompression, surgical outcomes

## Abstract

**Background/Objective**: Percutaneous transhepatic gallbladder drainage (PTGBD) is commonly used in patients with acute cholecystitis (AC) who are unfit for early laparoscopic cholecystectomy (LC). However, the efficacy, safety and long-term role of PTGBD remain debated. We aimed to evaluate the effectiveness and safety of PTGBD in managing AC, focusing on infection control, procedure-related complications, and need for secondary surgical intervention. **Methods**: We performed a single-center retrospective study including all patients who underwent PTGBD for AC from January 2018 to December 2023 at a tertiary care hospital. Patients were identified through an institutional database. Relevant clinical, procedural, and outcome data were extracted from electronic medical records. The primary outcome was infection control, defined as clinical and biochemical resolution of AC without the need for additional interventions beyond antibiotic therapy. Secondary outcomes included PTGBD-related complications, 30-day mortality, length of hospital stay, readmission rate, and the rate of subsequent LC. **Results**: A total of 105 patients were included (mean age 69.9 years; 63.8% male). Infection control was observed in 92.4% of patients following PTGBD. PTGBD-related complications occurred in 36.2%, mainly drain dislodgement and recurrent cholecystitis. Mortality was 4.8%. Delayed LC was performed in 80.9% of patients, with a 10.6% conversion rate and 16.5% postoperative complication rate. **Conclusions**: PTGBD is effective for infection control in high-risk AC patients unfit for immediate surgery. However, the complication rate and the frequent need for delayed LC underscore the importance of careful patient selection and standardized management strategies.

## 1. Introduction

Acute cholecystitis (AC) is a common and potentially severe inflammatory disease of the gallbladder, most frequently caused by obstruction of the cystic duct by gallstones [1,2]. It accounts for a significant proportion of emergency surgical admissions, and it is associated with substantial morbidity, particularly in elderly patients and those with multiple comorbidities [3,4,5,6]. For patients fit for surgery, the standard treatment for AC is early laparoscopic cholecystectomy (LC). Indeed, early LC has been shown to reduce complications, shorten hospital stay, and decrease healthcare costs when compared to delayed surgery or conservative management [5,7]. The Tokyo Guidelines (TG18) [8], World Society of Emergency Surgery (WSES) [9], and National Institute for Health and Care Excellence (NICE) [10] all recommend early LC as the treatment of choice when performed within 72 h of symptom onset, as this approach is associated with lower rates of complications, such as gangrenous cholecystitis, perforation, and biliary peritonitis.

However, a subset of patients with AC present with significant comorbidities, advanced disease stages, or hemodynamic instability, rendering them high-risk candidates for immediate surgical intervention [11,12,13]. In these cases, nonoperative management, including antibiotic therapy and percutaneous transhepatic gallbladder drainage (PTGBD), is often considered as an alternative or bridging strategy [3,8,11,12,14]. PTGBD, performed under ultrasound (US) or computed tomography (CT) guidance, leads to decompression of the inflamed gallbladder, rapid infection control, and potential avoidance of emergency surgery. This approach is particularly relevant for patients with severe sepsis, advanced cardiac or respiratory disease, or those under anticoagulant therapy, where surgical intervention carries an elevated perioperative risk [15,16,17]. Despite its widespread use, PTGBD remains a topic of debate due to inconsistencies in patient selection criteria and varying rates of procedural complications and uncertainties regarding its long-term efficacy. While some studies report high success rates in achieving infection control with PTGBD, concerns persist regarding recurrence of AC, drain-related complications (e.g., bile leaks, hemorrhage, drain dislodgement), and the potential for prolonged hospitalization [18,19,20,21]. Moreover, the role of PTGBD as a definitive treatment versus a bridging procedure remains unclear. A proportion of patients ultimately require delayed LC, which has been associated with higher conversion rates to open surgery, longer operative times and higher morbidity compared to early LC.

The decision to perform PTGBD is further complicated by a lack of standardized protocols regarding the timing of elective LC after drainage. While some reports suggest that interval LC should be performed within 6 to 8 weeks post-drainage to minimize complications, others indicate that prolonged delay increases the risk of adhesions, intraoperative difficulties and bile duct injuries [21,22,23,24]. Additionally, a proportion of patients treated with PTGBD never undergo definitive surgery, either due to persistent surgical contraindications or due to patient preference, raising concerns about long-term outcomes such as recurrent biliary events, chronic inflammation, and gallbladder malignancy [21,24,25,26].

Given these uncertainties, a better understanding of the clinical outcomes associated with PTGBD is essential to refine treatment algorithms for high-risk patients with AC. This study aims to evaluate the effectiveness and safety of PTGBD in a cohort of patients treated at a tertiary care center, with a focus on infection control, procedural complications, and the need for subsequent surgical intervention. By analyzing real-world data over a six-year period, we seek to clarify the role of PTGBD in AC management and provide evidence to guide decision-making for patients in whom immediate LC is not feasible.

## 2. Materials and Methods

### 2.1. Patients

Eligible patients were adults (≥18 years) with a diagnosis of AC, confirmed by clinical presentation, laboratory findings, and imaging, who underwent PTGBD after being considered high-risk candidates for early LC based on clinical evaluation. Patients with chronic cholecystitis, gallbladder malignancy, or a history of PTGBD prior to the study period were excluded, as were those with incomplete medical records or refusal to provide consent. No pregnant women or minors were included in the study cohort.

### 2.2. Ethical Aspects

The study protocol was approved by the local ethic committee (CER VD 2024-00311) on 26 June 2024. All patient data were coded, securely stored, and access was restricted to study investigators to ensure confidentiality and compliance with data protection regulations.

### 2.3. Study Outcomes

The primary outcome was infection control, defined as the resolution of clinical symptoms and normalization of inflammatory markers without the need for additional interventions beyond antibiotic therapy. Secondary outcomes comprised PTGBD-related complications, including procedure failure, bleeding, septic shock after drainage, drain dislodgement or obstruction, bile leakage, peritonitis, stone migration, and persistent or worsening cholecystitis. Additional endpoints included 30-day mortality, length of hospital stay, readmission within 30 days, and the need for intensive care unit admission. For patients who subsequently underwent delayed LC, surgical outcomes were assessed, including time to surgery, conversion to open cholecystectomy, operative time, and postoperative complications. In patients managed conservatively, recurrence of AC and the need for repeat drainage were recorded.

### 2.4. Study Design

This cohort study was conducted at the Cantonal Hospital of Fribourg in Switzerland, a tertiary care center and included retrospectively all patients who underwent PTGBD for AC from January 2018 to December 2023. Data were obtained from routine clinical care, including patient records, operative reports. and follow-up documentation. All patients were managed within the Department of Surgery by visceral surgeons trained in emergency general surgery, and not by subspecialized hepatopancreatobiliary or upper gastrointestinal surgeons.

### 2.5. PTGBD Procedure

**Figure 1:**
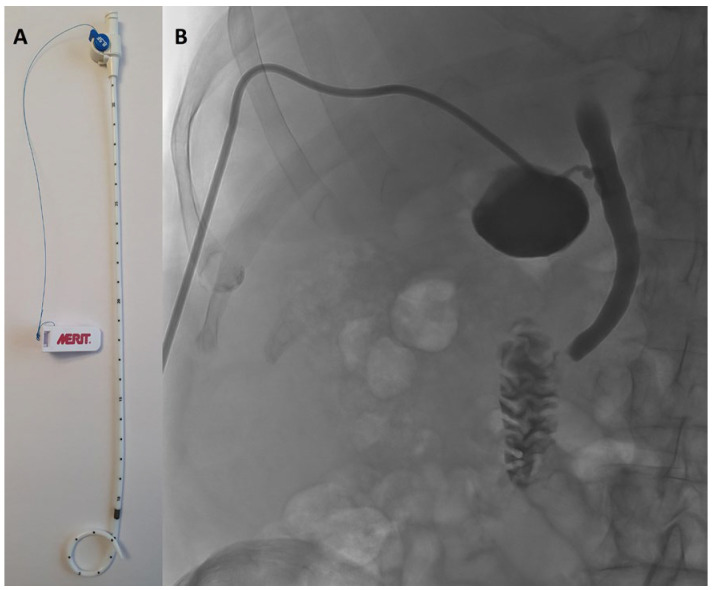
(**A**) 8.5 French pigtail catheter (Resolve^®^, Boston Scientific). (**B**) confirmation of the correct position of the drain by contrast injection and imaging.

PTGBD was performed under sterile conditions by an interventional radiologist using either US or CT guidance, depending on anatomical considerations and operator preference. PTGBD was done under local anesthesia and/or sedation according to patient comfort and cooperation. Under imaging guidance, a transhepatic approach accessed the gallbladder, followed by placement of an 8.5 French pigtail catheter (Re-solve^®^, Merit Medical Systems Inc., 1600 West Merit Parkway, South Jordan, UT, USA) for continuous drainage. Correct positioning was confirmed by contrast injection and imaging (Figure 1).

All procedures aimed to achieve immediate decompression of the gallbladder. Post procedural care included monitoring for complications, securing the drain, and ensuring adequate bile output. In addition to drainage, intravenous antibiotic therapy was also started in all patients as soon as the diagnosis of AC was made.

### 2.6. Follow-Up

A follow-up cholecystogram or a drain clamping test was not performed systematically but was left to the discretion of the treating physicians based on clinical and biochemical evolution. If a delayed LC was planned, the drain was left in place until surgery. In patients who did not undergo delayed LC, the decision to retain or remove the drain was individualized and based on clinical judgment.

### 2.7. Statistical Analysis

Continuous variables were expressed as mean with standard deviation (SD) or as median with interquartile range (IQR), depending on data distribution. Normality of continuous variables was assessed using the Shapiro–Wilk test before applying parametric or nonparametric statistical tests. Categorical variables were presented as frequencies and percentages. Univariate analysis was performed with Fisher’s exact test and Student’s t-test. Multivariate logistic regression was performed with the logistf package using Firth penalized logistic regression to identify risk factors predicting PTGBD failure and the need for subsequent surgery. A significance threshold of *p* < 0.05 was applied. All analyses were performed using Rstudio, (version 2024.09.0+375, Posit Software, PBC. Boston, MA, USA).

## 3. Results

The study included 105 patients. Patients’ demographics and clinical characteristics are described in Table 1. The majority had significant comorbidities (68.57%) and were male (63.8%). With regard to antithrombotic treatment, 28.57% of patients were receiving anticoagulant or double antiplatelet therapy. Immunosuppressive therapy was used by 8.57% of the cohort. The distribution of American Society of Anesthesiologists physical status scores (ASA score) reflected significant surgical risk, with 60% of patients classified as ASA score III or IV. 34.29% of patients are obese (BMI > 30 kg/m^2^).

**Figure 2:**
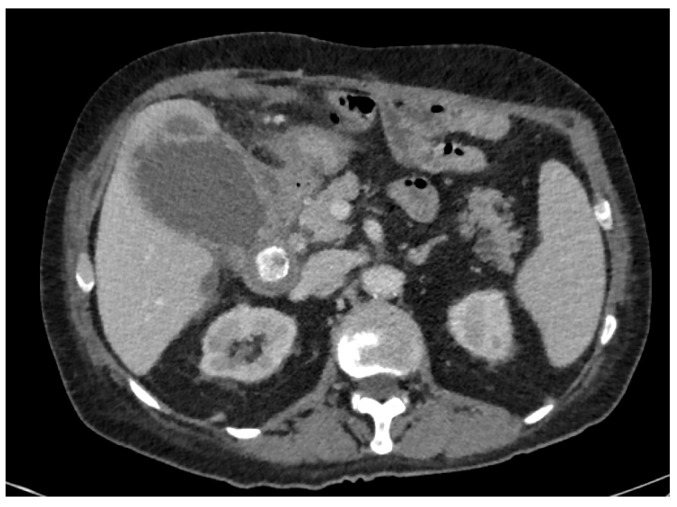
Initial CT scan in a 73-year-old man, known for ischemic and rhythmic heart disease anticoagulated with Apixaban, with an ASA score = 3, presenting pain for 2 weeks, with a significant inflammatory syndrome (CRP = 251 mg/L).

Among the 105 patients included in the study, the main reasons for not performing early LC were: symptom duration greater than 3 days according to internal departmental guidelines (*n* = 47, 44.76%), severe inflammatory status (*n* = 23, 21.90%) (Figure 2), and anticoagulant or dual antiplatelet therapy (*n* = 17, 16.19%). Additional reasons included significant comorbidities (*n* = 14), patient refusal (*n* = 3), and one case of suspected colobiliary fistula. Regarding the indications for PTGBD realization, the most common were worsening inflammatory markers despite appropriate antibiotic therapy (*n* = 50, 47.62%) and severe inflammatory status (C-reactive protein level > 300 mg/dL or sepsis) (*n* = 37, 35.24%). Less frequent indications included gallbladder perforation (*n* = 9) and gallbladder hydrops (*n* = 3). In six cases, the indication for PTGBD realization were not specified (Table 1).

PTGBD was successfully performed in nearly all patients with an overall success rate of 99.1%. The only failed attempt was due to patient agitation, requiring a second attempt under general anesthesia the following day, which was successful. The procedure was performed under local anesthesia in 97.1% of cases, thereby minimizing the need for advanced anesthetic support. PTGBD was typically carried out shortly after admission (median delay from admission to drainage = 1 day), using standardized equipment and predominantly ultrasound guidance (83.81%). Details of the PTGBD procedure, including imaging modality, anesthesia type, timing, drain specifications, and technical success rate, are summarized in Table 2.

### 3.1. Primary Outcome: Infection Control

Clinical and biochemical resolution of AC was achieved with PTGBD in 97 out of 105 patients (92.38%). Five patients died from septic shock with multi-organ failure despite appropriate drainage and antibiotic treatment. Three patients experienced severe complications after initial PTGBD. Of those, one required repeat drainage and emergency LC due to febrile relapse after stopping antibiotics. Another developed hemorrhagic shock from cystic artery injury, leading to percutaneous embolization and subsequent emergency LC for gallbladder necrosis. The third one was admitted to Intensive Care Unit (ICU) with septic shock and underwent emergency surgery for biliary peritonitis, requiring conversion to open surgery due to extensive inflammation and morbid obesity.

### 3.2. Risk Factors Associated with Infection Control After PTGBD

In univariate analysis, older age and chronic kidney disease were associated with reduced infection control after PTGBD (Table 3). In multivariate analysis, only advanced age (OR 0.84, 95% CI [0.73;0.95], *p* = 0.015) negatively impacted the effectiveness of percutaneous drainage, while chronic kidney disease (OR 0.16, 95% CI [0.03;1.06], *p* = 0.058) was borderline but not significant.

### 3.3. Secondary Outcomes

A total of 38 patients (36.19%) experienced at least one complication after PTGBD, with 50 complications reported overall. The most frequently observed adverse events were drain dislodgement (22%), recurrent AC (16%), stone migration (12%), and septic shock after drainage (10%). According to the Clavien–Dindo classification [27], complications were mostly of moderate to severe intensity: Grade 3, 4 and 5 represented 50%, 18% and 10% of complications, respectively (Table 4). Univariate analysis identified only ASA score > 2 (OR: 0.26, 95% CI [0.09;0.69], *p* = 0.0036) as significantly associated with mortality following PTGBD. This association, however, was not confirmed in multivariate analysis.

As clinical impact, the mean hospital stay was approximately 10 days (Table 5). ICU admission was required in nearly one-quarter of patients (23.81%). The combined in-hospital and 30-day mortality rate was 4.76%; all deaths were attributed to failure to control infection, resulting in septic shock and transition to comfort care, as per patient or family wishes. Univariate analysis identified only age (*p* = 0.0062) as significantly associated with mortality following PTGBD. This association, however, was not confirmed in multivariate analysis.

The 30-day readmission rate was 15.24% (*n* = 16), with one patient requiring two separate readmissions. The most frequent reason of readmission was recurrent AC (*n* = 6, 37.5%), followed by drain-related complications (2 obstruction and 2 dislodgement, 25%). Less common causes included stone migration and bleeding (*n* = 2 each, 12.5%). Two readmissions (12.5%) were due to non-surgical causes: global deconditioning and acute renal failure. Univariate logistic regression identified chronic kidney disease as the only significant predictor of 30-day rehospitalisation after PTGBD (OR = 0.25, 95% CI [0.06;1.14], *p* = 0.038). This association was not confirmed in multivariate analysis.

Late readmissions (>30 days) occurred in 10 patients (9.5%), with recurrent AC again being the leading cause (*n* = 3, 30%). Additional causes included drain-related complications: drain dislodgement, obstruction, or perforation (*n* = 1 each, 10%). The remaining four cases (40%) were attributed to unrelated medical conditions, such as paraneoplastic disseminated intravascular coagulation, mesenteric ischemia, cardiac decompensation, and autoimmune thrombocytopenia.

### 3.4. Surgical Outcomes in Patients Undergoing Delayed Laparoscopic Cholecystectomy

Among the 105 patients initially treated with percutaneous gallbladder drainage, 85 (80.9%) subsequently underwent delayed laparoscopic cholecystectomy (Table 6). Cholecystectomy was performed nearly 10 weeks after primary hospitalization (mean of 67 +/− 38.8 days) and an average of 65 days (SD 38.7 days) from PTGBD. Hospitalization was brief with a mean length of stay of 4.6 days (SD 4.5 days). The mean operative time was 2 h and 21 min, ranging from 50 min to over 5 h, reflecting variable technical difficulty.

Conversion to open surgery was required in 9 patients (10.5%). Adhesions were the leading cause of conversion (44.4%), followed by significant inflammatory changes (33.3%). In two cases (22.2%), conversion was prompted by intraoperative complications: one case of uncontrolled bleeding and one of diaphragmatic injury. Another conversion was due to the need for a concurrent surgical procedure requiring laparotomy (abdominal aortic aneurysm repair).

Postoperative complications occurred in 14 patients (16.3%), with a total of 19 adverse events. Ten of these complications (52.6%) were severe (Clavien–Dindo ≥ 3b). The most frequent issues were surgical site and wound infections (*n* = 4 each). Other complications included biliary leakage (*n* = 2), postoperative bleeding (*n* = 2), pancreatitis secondary to choledocholithiasis (*n* = 1), cardiogenic shock (*n* = 2), pneumonia (*n* = 1), portal vein thrombosis (*n* = 1) and global deconditioning requiring rehospitalization in geriatrics (*n* = 1). There was one postoperative death related to pneumonia in an elderly patient.

In this cohort, the interval between PTGBD and surgery was not associated with an increased risk of conversion to open surgery (mean difference: 12.98 days; 95% CI [−31.6; 57.5]; *p* = 0.51) or with postoperative complications (mean difference: 2.33 days; 95% CI [−12.6;17.2]; *p* = 0.74), suggesting that surgical timing did not impact short-term outcomes. Currently, the optimal timing for LC after PTGBD is not clearly defined in the literature, although a 6-week threshold is frequently reported. We performed a subgroup analysis comparing patients who underwent LC within 6 weeks of PTGBD to those operated on after 6 weeks. This analysis did not reveal any significant difference in terms of conversion to laparotomy (OR = 1.65, 95% CI [0.19–14.36], *p* = 0.65) or occurrence of complications (OR = 3.19, 95% CI [0.38–26.5], *p* = 0.282).

### 3.5. Outcomes in Patients Managed Conservatively

Excluding the two patients who underwent early cholecystectomy and the five patients who died during the initial hospitalization, 13 patients (13.40%) did not receive delayed cholecystectomy and were managed conservatively. The main reasons for non-operative management were significant comorbidities precluding surgical intervention in 7 cases (53.85%) and death prior to scheduled surgery in 3 patients (23.08%). It should be noted that these three deaths were not linked to gallbladder pathology. Last, two patients (15.38%) were diagnosed with acalculous cholecystitis, and one patient (7.69%) was lost to follow-up.

Of the 10 patients managed conservatively and alive, drain removal was successful in 9 cases (90%). However, recurrence of AC occurred in 3 patients (30%), and 4 patients (40%) required reinsertion of a drain (three due to recurrence of AC, one due to choledocholithiasis with impossibility of performing ERCP because of severe comorbidities). Two patients (20%) eventually underwent delayed cholecystectomy: one, two years later because of cholangitis caused by stone migration; the other during surgery for abdominal aortic aneurysm repair.

## 4. Discussion

In this single-center retrospective cohort study, we evaluated the role, outcomes, and limitations of PTGBD in the management of AC among patients deemed unfit for early LC. Our findings describe favorable outcomes with PTGBD, suggesting that it may serve as an effective bridging or temporizing strategy in selected high-risk patients, consistent with previous reports [28]. Nevertheless, the procedure was associated with a non-negligible rate of complications.

The primary outcome, infection control following PTGBD, was achieved in the majority of patients, with only few requiring early surgical intervention or progressing to septic shock and death. In multivariate analysis, advanced age emerged as the only independent predictor of treatment failure. This finding is consistent with prior studies, which have highlighted the impact of frailty and reduced physiological reserve on the outcomes of acute cholecystitis management. Chronic kidney disease also showed a strong trend toward significance, suggesting a clinically relevant association even though statistical significance was not reached. This lack of significance is most likely attributable to the limited sample size. Another important consideration is the role of cardiovascular comorbidities in selecting patients for PTGBD. In our study, “cardiovascular disease” was recorded as a broad category, without systematic distinction between ischemic heart disease and congestive heart failure. This represents a limitation of our retrospective dataset. Nevertheless, several patients with advanced heart failure were indeed managed with PTGBD due to contraindications for general anesthesia. This observation underscores the clinical relevance of severe cardiac failure as a key indication for PTGBD and highlights the importance of future studies differentiating between cardiac subgroups to better define outcomes and refine patient selection.

Complications following PTGBD occurred in over one-third of cases, drain dislodgement, recurrent AC, stone migration and septic shock after drainage were among the most common adverse events. Risk analysis did not identify any factors associated with post-drainage complications. These complications may prolong hospitalization and lead to unplanned reinterventions, underscoring the procedural risks despite its minimally invasive nature.

Thirty-day readmission occurred in 16% of patients, predominantly due to recurrent cholecystitis or drain-related issues. The overall in-hospital and 30-day mortality rate was 4.8%, with all deaths attributed to failure of infection control. Risk analysis did not identify any factors associated with thirty-day readmission or mortality.

Delayed laparoscopic cholecystectomy was feasible in over 80% of patients and was generally performed within 10 weeks of drainage. Operative outcomes were within expected ranges. However, a conversion to open surgery was required in 10.6% of cases, primarily due to dense adhesions or chronic inflammatory changes. Postoperative complications occurred in 16.5% of patients, including several severe events. Moreover, the mean operating time is relatively long (over than 2 h). These findings are consistent with previous studies showing that delayed LC is technically feasible but can be surgically challenging, particularly in patients with complex anatomy or systemic frailty [2,7,14,15,21,24]. These results underline the importance of preoperative risk stratification in patients undergoing interval cholecystectomy. Older age and comorbidities should be considered key factors when evaluating the risk of postoperative complications in this high-risk population [5]. While the design of our study does not allow for this comparison, it would be valuable to assess, in future studies, differences in mean operative time, conversion rates, and complication rates between LC performed after PTGBD and LC performed during the acute phase.

Importantly, 13.4% of patients did not undergo cholecystectomy after PTGBD. Contributing factors included early mortality and persistent medical ineligibility. Among these patients, recurrence of cholecystitis and the need for repeat drainage were observed, suggesting that PTGBD may be insufficient as a definitive therapy in some cases. Although drain removal was achieved in most survivors, the long-term efficacy of conservative management remained limited. Another important aspect to consider is the risk of underlying gallbladder neoplasia that may remain undiagnosed in patients who do not undergo cholecystectomy. This concern is particularly relevant given that diabetes, obesity, and acalculous cholecystitis are recognized risk factors for gallbladder malignancy [29]. These elements should therefore be carefully weighed in the decision-making process when opting against definitive cholecystectomy.

The timing of delayed LC is a subject of ongoing debate. Some guidelines advocate for surgery within 6 to 8 weeks after drainage to minimize operative difficulty, while others suggest tailoring timing based on patient recovery and comorbidities. In our cohort, no association was observed between the timing of surgery after PTGBD and the rates of conversion or postoperative complications. Our results are also in line with the recent meta-analysis by Sadaka et al. [30], which synthesized global evidence on PTGBD for AC. This study confirmed high rates of infection control and technical success, but also emphasized the significant morbidity associated with drain-related complications and the frequent need for interval LC. In particular, Sadaka et al. reported that delayed LC after PTGBD was associated with better outcomes compared with PTGBD alone, and suggested an optimal timing of 8 to 13 weeks after drainage. These findings are consistent with our observation that delayed LC was feasible in over 80% of patients, though often technically challenging, while patients managed conservatively experienced higher rates of recurrence and repeat interventions. By providing detailed risk factor analysis and outcome data in a consecutive high-risk cohort, our study adds precision to the broader conclusions of this meta-analysis and supports PTGBD as a temporizing rather than definitive therapy in this setting.

This study has several strengths, including a well-defined cohort, detailed procedural and outcome data, and rigorous statistical analysis. However, several limitations must be acknowledged. The retrospective design introduces inherent biases, including selection bias and variability in treatment decisions. Additionally, the single-center nature of the study may limit the generalizability of our findings. In particular due to local departmental guidelines excluding the performance of an early LC in the event of symptoms evolving for more than 3 days. This institutional protocol, while intended to minimize operative risk in advanced cases, inevitably influenced patient selection and may have led to a higher proportion of patients undergoing PTGBD. Such center-specific practices introduce potential bias and limit the external generalizability of our results, as institutions with different management strategies may report different outcomes. Our findings should therefore be interpreted with caution when applied to centers that routinely perform early LC in patients with longer symptom duration. It should be noted that among the 47 patients excluded due to a symptom duration of more than 3 days, the majority had comorbidities (95.74% with an ASA score > 2) or presented another contraindication to immediate cholecystectomy (including 10.64% of patients on anticoagulation therapy). Another major limitation is the absence of a comparator group (e.g., patients managed with early LC, conservative therapy alone, or alternative drainage methods such as EUS-guided drainage). Without such a control group, our results can only be interpreted in a descriptive manner, and causal inferences cannot be drawn. While our findings provide valuable real-world outcome data, they cannot determine the relative efficacy or safety of PTGBD compared with other treatment strategies. Finally, while we adjusted for key clinical variables, unmeasured confounders may have influenced outcomes, particularly regarding surgical decision-making and long-term prognosis.

Despite these limitations, our findings really contribute to the growing evidence base on PTGBD, supporting its role in managing high-risk AC patients. It emphasizes the importance of individualized care pathways and multidisciplinary evaluation.

## 5. Conclusions

PTGBD is a valuable therapeutic option for patients with AC who are not candidates for early LC. In our cohort, PTGBD demonstrated high efficacy in achieving infection control and was associated with a low 30-day mortality rate. However, we should consider that PTGBD carries a non-negligible risk of complications.

Delayed cholecystectomy remains feasible in the majority of cases and offers satisfactory surgical outcomes, although the higher risk of conversion and postoperative complications underscores the need for careful patient selection. For patients managed conservatively, recurrence of symptoms and the need for re-intervention remain frequent.

Overall, our findings provide descriptive, real-world data that highlight the importance of tailored therapeutic strategies and multidisciplinary evaluation to optimize outcomes in high-risk populations with acute cholecystitis. Further prospective and comparative studies are needed to better define the role of PTGBD in AC management and establish evidence-based guidelines for its use.

## Figures and Tables

**Table 1:** Patient Demographics and Clinical Characteristics. Baseline demographics, comorbidities and clinical presentation of the included patients; severity grading of acute cholecystitis (AC) according to the Tokyo Guidelines (TG18); reasons for ineligibility for early laparoscopic cholecystectomy and indications for percutaneous transhepatic gallbladder drainage (PTGBD) realization. BMI = Body Mass Index. ASA Score = American Society of Anesthesiologists score.

	N	%	Mean ± SD
Amount of Patients	105	100%	
Age (years)			69.88 ± 12.98
Sex			
Male	67	63.81%	
Female	38	36.19%	
Comorbidity			
None	33	31.43%	
Diabetes	30	28.57%	
Chronic Kidney Disease	15	14.29%	
Cardiovascular Disease	43	40.95%	
Pulmonary Disease	16	15.24%	
BMI > 30 kg/m^2^	36	34.29%	
Anticoagulant treatment	22	20.95%	
Double antiplatelet treatment	8	7.62%	
Immunosuppressive Therapy	9	8.57%	
ASA Score			
1	7	6.67%	
2	35	33.33%	
3	53	50.48%	
4	10	9.52%	
Time from symptom onset to hospital admission (days)			3.77 ± 3.89
Grade according to TG18 classification			
Grade I	13	12.38%	
Grade II	68	64.76%	
Grade III	24	22.86%	
Lithiasic			
Yes	98	93.33%	
No	7	6.67%	
Causes of non-performance of early LC			
Anticoagulant or double antiplatelet therapy	17	16.19%	
Symptoms lasting > 3 days	47	44.76%	
Severe inflammatory status	23	21.90%	
Patient refusal	3	2.86%	
Comorbidities	14	13.33%	
Other	1	0.95%	
Indications for PTGBD realization			
Worsening inflammatory markers despite antibiotic therapy	50	47.62%	
Severe inflammatory status	37	35.24%	
Gallbladder hydrops	3	2.86%	
Gallbladder perforation	9	8.57%	
Not specified	6	5.71%	

**Table 2:** Percutaneous transhepatic gallbladder drainage procedure characteristics. CT = Computed Tomography.

	N	%	Mean ± SD	Median
Imaging modality for drain placement				
Ultrasound-guided	88	83.81%		
CT-guided	17	16.19%		
Type of anesthesia				
Local anesthesia	102	97.14%		
Sedation	3	2.86%		
General anesthesia	0	0.00%		
Delay from admission to drainage (days)			2.61 (±4.10)	1.0
Technical success rate	104	99.05%		
Failed attempt	1 case due to agitation; successful on second attempt under general anesthesia			

**Table 3:** Univariate and multivariate analysis between risk factors and infection control after PTGBD. For univariate analysis: used Student’s t-test for age and Fisher’s exact test for all other variables. For multivariate analysis: used firth logistic regression (Likelihood ratio test = 23.66, *p* = 0.014. Wald test = 28.10, *p* = 0.0031). BMI = Body Mass Index. ASA Score = American Society of Anesthesiologists score. ND = not determined (Odds ratio not calculable for ASA score > 2 due to complete data separation).

Risk Factor	Odds Ratio	Low CI	High CI	*p*-Value
Univariate analysis				
Age				0.0017
Sex	1.19	1.07	1.32	0.12
Diabetes	1.28	0.24	6.72	0.77
BMI > 30 kg/m^2^	1.16	0.26	5.17	0.84
Chronic Kidney Disease	7.82	1.71	35.08	0.0081
Cardiovascular Disease	2.59	0.58	11.46	0.21
Pulmonary Disease	1.28	0.15	11.17	0.82
ASA score > 2	ND	ND	ND	0.99
Antithrombotic treatment	2.73	0.64	11.72	0.18
Immunosuppressive Therapy	1.59	0.17	14.58	0.68
Multivariate analysis				
Age	0.84	0.73	0.95	0.015
Sex	1.22	0.52	2.95	0.18
Diabetes	1.77	0.56	2.98	0.56
BMI > 30 kg/m^2^	0.33	0.18	1.42	0.40
Chronic Kidney Disease	0.16	0.03	1.06	0.058
Cardiovascular Disease	1.00	0.18	1.82	0.99
Pulmonary Disease	2.09	0.24	4.48	0.62
ASA score > 2	0.07	0.18	3.29	0.07
Antithrombotic treatment	1.16	0.21	2.27	0.88
Immunosuppressive Therapy	1.59	0.44	4.44	0.75

**Table 4:** Percutaneous transhepatic gallbladder drainage related complications.

	N (Tot = 105)	%
Patients with at least one complication	38	36.19%
Total number of complications	50	
Type of complication		
Septic shock after drainage	5	10.00%
Drainage failure	1	2.00%
Bile leak	1	2.00%
Bleeding	2	4.00%
Drain dislodgement	11	22.00%
Drain obstruction	4	8.00%
Peritonitis	2	4.00%
Stone migration	6	12.00%
Recurrent cholecystitis	8	16.00%
Other	5	10.00%
Death	5	10.00%
Clavien–Dindo classification		
Grade 1	2	4%
Grade 2	4	8%
Grade 3a	18	36%
Grade 3b	12	24%
Grade 4a	5	10%
Grade 4b	4	8%
Grade 5	5	10%

**Table 5:** In-hospital outcomes and 30-day mortality rates. ICU = Intensive Care Unit.

	N	%	Mean ± SD	Median
Length of hospital stay (days)			10.25 ± 7.25	8
Need for ICU admission	25	23.81%		
30-day readmission	16	15.24%		
Causes of 30-day readmission				
Recurrent cholecystitis	6	37.50%		
Drain dislodgement	2	12.50%		
Drain obstruction	2	12.50%		
Stone migration	2	12.50%		
Bleeding	2	12.50%		
Other	2	12.50%		
>30-day readmission	10	9.5%		
Causes of >30-day readmission				
Recurrent cholecystitis	3	30%		
Drain dislodgement	1	10%		
Drain obstruction	1	10%		
Drain perforation	1	10%		
Other	4	40%		
In-hospital and 30-day mortality	5	4.76%		

**Table 6:** Surgical outcomes in patients undergoing delayed laparoscopic cholecystectomy.

	N	%	Mean ± SD	Range
Amount of patients	85	80.95%		
Time interval from PTGBD to surgery (days)			65.7 ± 38.77	
Operative characteristics				
Duration (min)			141 ± 58	50–304
Conversion to open surgery	9	10.59%		
Cause of conversion:				
Adhesions	4			
Bleeding	1			
Severe inflammation	3			
Other procedure requiring laparotomy	1			
Intraoperative complications	2	2.35%		
Diaphragm injury	1			
Uncontrollable bleeding	1			
Postoperative complications				
- Patients with at least one complication	14	16.47%		
- Total number of complications	19			
- Clavien–Dindo classification				
Grade 1	2	10.53%		
Grade 2	3	15.79%		
Grade 3a	4	21.05%		
Grade 3b	7	36.84%		
Grade 4a	1	5.26%		
Grade 4b	1	5.26%		
Grade 5	1	5.26%		
- Type of postoperative complication				
Deaths	1			
Bleeding	2			
Bile leak	2			
Scar infection	4			
Surgical site infection	4			
Other	6			
Length of hospital stay (days)			4.65 ± 4.52	

## Data Availability

The raw data supporting the conclusions of this article will be made available by the authors on request.

## Abbreviations

The following abbreviations are used in this manuscript:

AC	Acute cholecystitis
LC	Laparoscopic cholecystectomy
TG18	Tokyo Guidelines 2018
WSES	World Society of Emergency Surgery
NICE	National Institute for Health and Care Excellence
PTGBD	Percutaneous transhepatic gallbladder drainage
US	Ultrasound
CT	Computed tomography
BMI	Body mass index
ASA	American Society of Anesthesiologists
ICU	Intensive Care Unit
ERCP	Endoscopic retrograde cholangiopancreatography
CRP	C-reactive protein
SD	Standard deviation
IQR	Interquartile range
OR	Odds ratio
95% CI	95% confidence interval
